# Effects of Xuefu Zhuyu oral liquid as adjunctive treatment for stable angina: a randomized controlled trial

**DOI:** 10.3389/fmed.2026.1787481

**Published:** 2026-04-29

**Authors:** Wencong Cao, Shaojun Liao, Li Zhou, Geng Li, Junwen Jiang, Weihui Lv, Bo Dong, Li Liu, Jie Zhang, Yanchun Wang, Wenwei Ouyang, Yi Du, Zehuai Wen

**Affiliations:** 1 The Second Clinical College of Guangzhou University of Chinese Medicine, Guangzhou, China; 2 Vincent V.C. Woo Chinese Medicine Clinical Research Institute, School of Chinese Medicine, Hong Kong Baptist University, Kowloon, Hong Kong SAR, China; 3 Chinese EQUATOR Centre, Kowloon, Hong Kong SAR, China; 4 Second Affiliated Hospital of Guangzhou University of Chinese Medicine, Guangzhou, Guangdong, China; 5 Center for Clinical Research, Guangdong Provincial Hospital of Chinese Medicine, Guangzhou, Guangdong, China; 6 Guangdong Provincial Key Laboratory of Clinical Research on Traditional Chinese Medicine Syndrome, Guangzhou, Guangdong, China; 7 State Key Laboratory of Dampness Syndrome of Chinese Medicine, Second Affiliated Hospital of Guangzhou University of Chinese Medicine, Guangzhou, Guangdong, China; 8 The First Affiliated Hospital of Liaoning University of Traditional Chinese Medicine, Shenyang, China; 9 The Second Affiliated Hospital of Liaoning University of Traditional Chinese Medicine, Shenyang, China; 10 The First Affiliated Hospital of Heilongjiang University of Chinese Medicine, Harbin, China; 11 The First Hospital of China Medical University, Shenyang, China; 12 Shenyang Tenth People’s Hospital, Shenyang, China

**Keywords:** Chinese herbal formula, Chinese medicine, randomized controlled trial, stable angina, Xuefu Zhuyu oral liquid

## Abstract

**Background:**

Stable angina (SA) remains a major cause of global disability. Xuefu Zhuyu oral liquid (XZOL), a traditional Chinese medicine, is used for SA in China, but rigorous evidence of its efficacy is lacking. We aimed to evaluate the efficacy and safety of XZOL as an adjunctive therapy for patients with SA.

**Methods:**

We conducted a multicenter, randomized, double-blind, placebo-controlled trial across six hospitals in China. Between June 2020 and June 2022, eligible patients with SA were randomly assigned (1:1) to receive either XZOL or a matching placebo for 12 weeks, in addition to standard antianginal therapy. All participants were followed for an additional 12 weeks. The primary outcome was the change in average angina pain intensity from baseline to week 12, measured on a 10-cm visual analog scale (VAS).

**Results:**

Of 263 patients screened, 148 were included in the full analysis set (74 per group). At week 12, patients receiving XZOL showed a reduction in VAS pain scores compared to placebo (adjusted mean difference, –0.63; 95% CI, –1.12 to –0.14; *P* = 0.012). This effect was sustained at the 24-week follow-up (–0.47; 95% CI, –0.94 to –0.002; *P* = 0.049). Furthermore, the XZOL group had significantly lower use of rescue nitroglycerin at both week 12 (2.7% vs. 13.5%; *P* = 0.016) and week 24 (2.7% vs. 12.2%; *P* = 0.029). Adverse events were comparable between groups.

**Conclusion:**

Adjunctive treatment with XZOL for 12 weeks significantly reduced angina pain intensity and the need for rescue medication in patients with stable angina, with a favorable safety profile.

**Clinical trial registration:**

https://clinicaltrials.gov/, ChiCTR1900026899.

## 1 Introduction

Stable angina (SA), a clinical syndrome caused by myocardial ischemia, remains a significant global health concern. It is associated with a 3–4% annual risk of myocardial infarction or death and affects an estimated 9.6% of the adult population in China, imposing a substantial burden on both healthcare systems and patient quality of life (1, 2). Current guideline-directed medical therapy—including lifestyle modifications, pharmacotherapy, and revascularization—aim to alleviate symptoms, improve exercise tolerance, and reduce the risk of cardiovascular events (3). However, the efficacy of these treatments is often incomplete, with a considerable proportion of patients continuing to experience anginal symptoms and activity limitations despite optimal medical management (4). Moreover, the long-term use of conventional antianginal drugs can lead to tolerability issues and adverse effects (AEs), highlighting the need for effective complementary therapeutic strategies (5).

Chinese Medicine (CM) has emerged as a promising adjunctive approach for managing SA. A systematic review suggested that combining conventional treatment with CM for angina pectoris may offer benefits in symptom and serum cholesterol reduction, but the evidence is limited (6). More recently, a large-scale real-world study demonstrated that an integrated medicine approach significantly reduced cardiovascular events compared to conventional treatment alone; however, the study’s single-center design and the heterogeneity of the CM interventions limit the generalizability of these findings (7). Collectively, these studies indicate the therapeutic potential of CM but also underscore the ongoing controversy surrounding its clinical application, which stems from a lack of high-quality, definitive evidence.

Among the numerous herbal formulas, Xuefu Zhuyu decoction, a classical prescription, is traditionally used for conditions characterized by “Qi stagnation and Blood stasis” (QBS)—a CM diagnostic pattern that conceptually aligns with the blood flow obstruction and ischemic pain observed in SA (8). Xuefu Zhuyu oral liquid (XZOL) is a modern, standardized formulation of this decoction, manufactured under Good Manufacturing Practice (GMP) standards and approved by the China Food and Drug Administration (Approval No. Z10950063). It is composed of 11 herbs, including *Bupleuri Radix* (Chaihu), *Persicae Semen* (Taoren), *Paeoniae Radix Rubra* (Chishao), *Aurantii Fructus Immaturus* (Zhiqiao), *Rehmanniae Radix* (Shengdihuang), *Achyranthis Bidentatae Radix* (Niuxi), *Angelicae Sinensis Radix* (Danggui), *Carthami Flos* (Honghua), *Glycyrrhizae Radix et Rhizoma* (Gancao), *Platycodonis Radix* (Jiegeng) and *Chuanxiong Rhizoma* (Chuanxiong) (Supplementary Table 1 in Supplementary File 1).

The therapeutic rationale for XZOL in SA is supported by a growing body of preclinical evidence. A pharmacological study has demonstrated that key components, including *Chuangxiong Rhizoma, Carthami Flos, Rehmanniae Radix, Aurantii Fructus, and Glycyrrhizae Radix et Rhizoma*, substantially inhibit adenosine diphosphate (ADP)-induced platelet aggregation (9). Animal models have further revealed their multi-target effects, including anti-atherosclerotic actions, reduction of serum lipids and inflammation, repair of endothelial injury, and stabilization of atherosclerotic plaques—all of which are critical mechanisms in the pathophysiology of coronary artery disease (10–12). Although a meta-analysis of earlier, smaller trials suggested that the Xuefu Zhuyu formula is effective for angina pectoris (13), this evidence is undermined by significant methodological limitations. Therefore, a rigorously designed, large-scale, placebo-controlled trial is essential to definitively establish the efficacy and safety of XZOL. This study was designed to address that evidence gap.

## 2 Methods

### 2.1 Study design

This multicenter, double-blind, randomized, placebo-controlled trial was conducted between June 2020 and June 2022 at six sites ascross China. The study protocol was approved by the Ethics Committees at Guangdong Provincial Hospital of Chinese Medicine (Approval No. BF2019-175-01) and was registered in the Chinese Clinical Trial Registry (ChiCTR1900026899). The trial is reported in accordance with the Consolidated Standards of Reporting Trials (CONSORT) Extension for Chinese Herbal Medicine Formulas 2017 reporting guideline (14). The study protocol has been previously published (15). Written informed consent was obtained from all participants prior to enrollment.

### 2.2 Participants

This study enrolled patients aged 30–75 years who met the following inclusion criteria: (1) a confirmed diagnosis of SA secondary to coronary artery disease, according to the 2018 guidelines from the Chinese Society of Cardiology for stable ischemic heart disease (16) and the 2002 AHA/ACC guideline update on the management of chronic stable angina (17); (2) a CM diagnosis of the QBS, as assessed by a validated diagnostic instrument (18); (3) angina severity graded as I, II, or III according to the Canadian Cardiovascular Society (CCS) grading system (19); (4) objective evidence of coronary artery disease, defined as at least one of the following: (a) a positive exercise treadmill test, (b) a positive radionuclide exercise test, (c) history of myocardial infarction ( > 3 months prior), (d) history of percutaneous coronary intervention (PCI) ( > 12 months prior), or (e) coronary angiography or CT angiography showing ≥ 50% stenosis in at least one major coronary artery or its main branches; (5) the frequency of angina attack between ≥ 2 times per week and ≤ 6 times per day; and (6) a self-reported pain score of ≥ 3 cm on a 10-cm visual analog scale (VAS) within the 2 weeks preceding enrollment.

Exclusion criteria included: (1) severe heart disease and other conditions known to cause non-cardiac chest pain; (2) poorly controlled hypertension; (3) planned coronary revascularization during the trial period; (4) severe hepatic or renal dysfunction, and severe primary diseases involving the respiratory, digestive, urinary, hematopoietic systems; (5) active malignancy; (6) severe anxiety or depression- defined as a score exceeding 59 on the Zung Self-Rating Anxiety Scale (20) or 62 on the Zung Self-Rating Depression Scale (21). A detailed list of all the inclusion and exclusion criteria is provided in Supplementary File 2 (Supplementary Tables 1–3).

### 2.3 Randomization and blinding

A center-stratified randomization sequence (block size of four) was generated using SAS version 9.2 (SAS Institute Inc., Cary, NC, United States). Randomization was managed by the Institute of Basic Research in Clinical Medicine (IBRCM) at the China Academy of Chinese Medical Sciences (Beijing, China). After confirming eligibility, participants were randomly allocated in a 1:1 ratio to receive either XZOL or a placebo via a central Interactive Web Response System (IWRS) managed by IBRCM.

Both the XZOL and the placebo were produced in oral liquid form by Jilin Aodong Yanbian Pharmaceutical Co., Ltd. (Jilin, China) in compliance with GMP standards. The placebo formulation- containing granulated sugar, brown sugar, honey, ginseng essence, bitters, natural food-grade pigments, and a food-grade preservative—was meticulously designed to match XZOL in color, taste, appearance, dosage, packaging, and labeling. Further details are provided in Supplementary File 1.

Allocation concealment was strictly maintained throughout the study. All parties involved, including participants, investigators, and the statistician, were blinded to group assignments, which IBRCM independently completed and managed.

### 2.4 Procedures

Following enrollment, all patients underwent antianginal therapy, including antihyperlipidemic agents, anticoagulants, β-blockers, angiotensin-converting enzyme (ACE) inhibitors, angiotensin II receptor blockers (ARBs) and calcium channel blockers (CCBs). The specific regimen was tailored to each patient’s clinical profile and comorbidities. In addition to this standard care, participants received either the XZOL or a matching placebo at a dosage of 20 mL per dose, three times daily over a 12-week period. Follow-up assessments were conducted by trained study personnel at weeks 2, 4, 8, and 12 (treatment phase) and at week 24 (post-treatment follow-up).

Sublingual nitroglycerin (0.5 mg) was provided as the rescue medicine for acute angina attacks. Participants were instructed to record the time, date, and dosage of any rescue medication used in a patient diary. Use of any other rescue medication was considered a protocol violation.

The study was supervised by an independent steering committee and a Data and Safety Monitoring Committee (DSMC). All reports of serious adverse events (SAEs) were promptly submitted to the ethics committee and the DSMC for review. Demographic characteristics, including sex, race and ethnicity, were initially documented on paper-based case report forms in reserved rooms. These data were subsequently entered into central electronic data capture (EDC) system developed by IBRCM.

### 2.5 Outcomes measurements

The primary outcome was the change in the average angina pain intensity from baseline to week 12, as measured by a 10-cm VAS. On this scale, 0 indicates “no pain” and 10 indicates “worst imaginable pain,” with lower scores representing better outcomes. Secondary outcomes, assessed from baseline through week 24, included: (1) the change in the average angina pain intensity; (2) frequency of angina attacks; (3) duration of angina attacks; (4) nitroglycerin dosage consumed; (5) angina severity based on CCS grading system for effort angina; (6) changes in the QBS pattern; (7) impact of angina on health status as measured by Seattle Angina Questionnaire (SAQ) (22); (8) quality of life assessed using the EuroQol-5-Dimensions-5-Level (EQ-5D-5L) (23); and (9) sleep quality assessed via the Pittsburgh Sleep Quality Index (PSQI) (24). Safety was assessed by documenting all AEs siazw and SAEs. The causality between the study intervention and reported AEs was determined using the World Health Organization–Uppsala Monitoring Centre (WHO-UMC) system for standardized case causality assessment (25).

### 2.6 Statistical analysis

Based on findings from a prior study (26), a sample size of 61 participants per group would be required to detect a superiority margin of 1.7 on the VAS for angina pain, assuming a standard deviation of 1.5 with a 2-tailed test, 90% statistical power and a significance threshold of *P* < 0.05. To account for a projected 20% drop-out, the final target enrollment was set at 152 participants (76 per group).

Efficacy outcomes were analyzed using a modified intention-to-treat (mITT) principle. The analysis population, referred to as the full analysis set (FAS), included all randomized patients who received at least one dose of the study intervention and provided at least one post-baseline efficacy assessment. Missing data for the primary outcome were handled using multiple imputation, and analyses were conducted on five imputed datasets (Supplementary File 3). Continuous variables were reported as mean with standard deviation (SD) and compared using either Student’s *t*-test or the Wilcoxon rank-sum test. Categorical variables were summarized as frequencies and percentages, and analyzed using the chi-square test, Fisher’s exact test or Mann-Whitney *U*-test, as appropriate.

The primary outcome—change in VAS score from baseline to Week 12—was analyzed using a generalized linear model, with adjustments for baseline VAS score, study center, and rescue medication use, and the interaction between center and treatment. Secondary outcomes were assessed for statistically significant differences from baseline to Week 24. The safety set (SS), which included all participants who received at least one dose of the intervention, was used for all safety analyses.

A key sensitivity analysis was conducted based on the per-protocol set (PPS), which included participants who completed the 12-week intervention and had no major protocol violations. Prespecified subgroup analyses were also conducted based on sex, age ( ≤ 65 vs. > 65 years), and CCS grade (I–II vs. III). All statistical tests were two-tailed, and a *P*-value < 0.05 was considered statistically significant. Analyses were carried out using SPSS 18.0 (IBM SPSS Inc., Armonk, NY, United States).

## 3 Results

**FIGURE 1:**
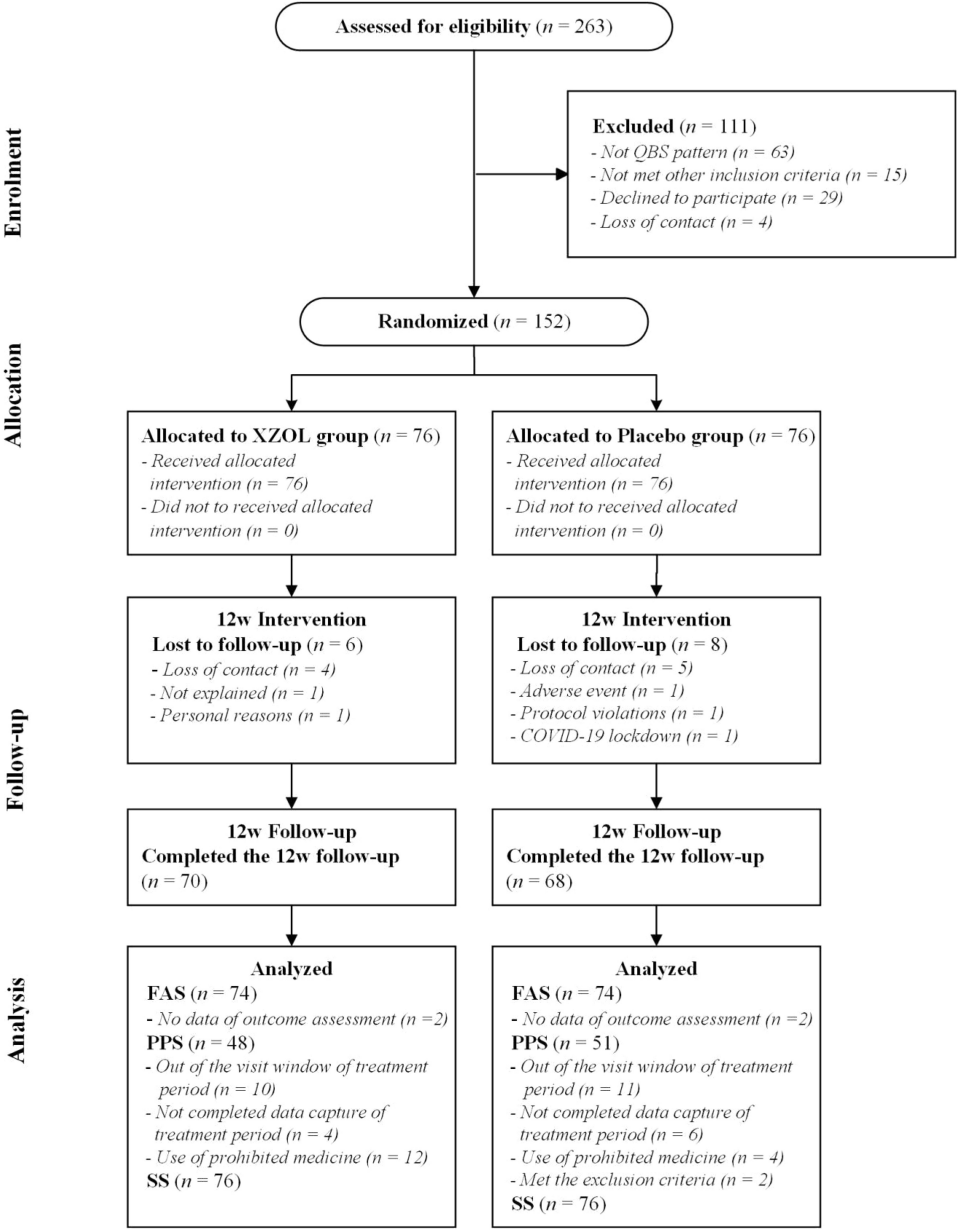
CONSORT study flow diagram. QBS, Qi stagnation and Blood stasis; XZOL, Xuefu Zhuyu oral liquid; FAS, full analysis set; PPS, per-protocol analysis set; SS, safety set.

Between June 2020 and June 2022, a total of 263 individuals were screened across six tertiary hospitals in China, of whom 152 were deemed enrolled and randomized. Four patients (2.6%, two in each group) were excluded from the FAS because they did not provide any post-baseline efficacy assessments. This left a final FAS of 148 patients (74 per group). A total of 99 participants (48 in the XZOL group, 51 in the placebo group) completed the 12-week treatment and the 12-week follow-up period (Figure 1). Baseline demographic and clinical characteristics are summarized in Table 1.

**TABLE 1:** Baseline characteristics (FAS).

Variable	Participants, no. (%) (*N* = 148)	
	XZOL group (*n* = 74)	Placebo group (*n* = 74)
Age, mean (SD), y	61 (8.97)	61 (8.61)
Sex, females	21 (28.4)	26 (35.1)
Anthropometry		
Height, mean (SD), cm	167.47 (7.03)	167.28 (6.93)
Weight, mean (SD), kg	71.36 (11.41)	70.18 (11.33)
Ethnicity, Han	72 (97.3)	72 (97.3)
Education		
High school diploma or less	62 (84.7)	62 (84.7)
Incomplete college education or higher	12 (16.2)	12 (16.2)
Family history, yes	66 (89.2)	63 (85.1)
Resting blood pressure, mean (SD), mm Hg		
Systolic	129.64 (9.74)	128.99 (9.82)
Diastolic	77.15 (8.08)	77.76 (9.38)
Participant smokes any cigarettes, yes	39 (52.7)	27 (36.5)
Participant consumes any alcoholic beverages, yes	24 (32.4)	16 (21.6)
Disease duration, mean (SD), months	43.90 (42.29)	41.28 (51.04)
Antianginal medications		
Nicorandil	0 (0)	1 (1.4)
Nitrates (nitroglycerin or isosorbide mononitrate)	6 (8.1)	8 (10.8)
Concomitant medications		
Beta-blockers	39 (52.7)	32 (43.2)
CCB	13 (17.6)	20 (27.0)
Anticoagulants	2 (2.7)	0 (0)
Statins	64 (86.5)	62 (83.8)
Statins dosage, mean (SD), mg	13.81 (8.0)	13.54 (8.1)
Low-intensity	1 (1.4)	0 (0)
Moderate-intensity	70 (94.6)	72 (97.3)
High-intensity	3 (4.1)	2 (2.7)
Antiplatelets	64 (86.5)	69 (93.2)
ACE inhibitor	4 (5.4)	4 (5.4)
ARB	13 (17.6)	14 (18.9)
Previous use of PCI	4 (5.4)	2 (2.7)
Previous use of coronary angiogram	52 (70.3)	54 (73.0)

FAS, full analysis set; SD, standard deviation; XZOL, Xuefu Zhuyu oral liquid; CCB, calcium channel blockers; ACE, angiotensin-converting enzyme; ARB, angiotensin II receptor blockers; PCI, percutaneous coronary intervention.

### 3.1 Primary outcome

**FIGURE 2:**
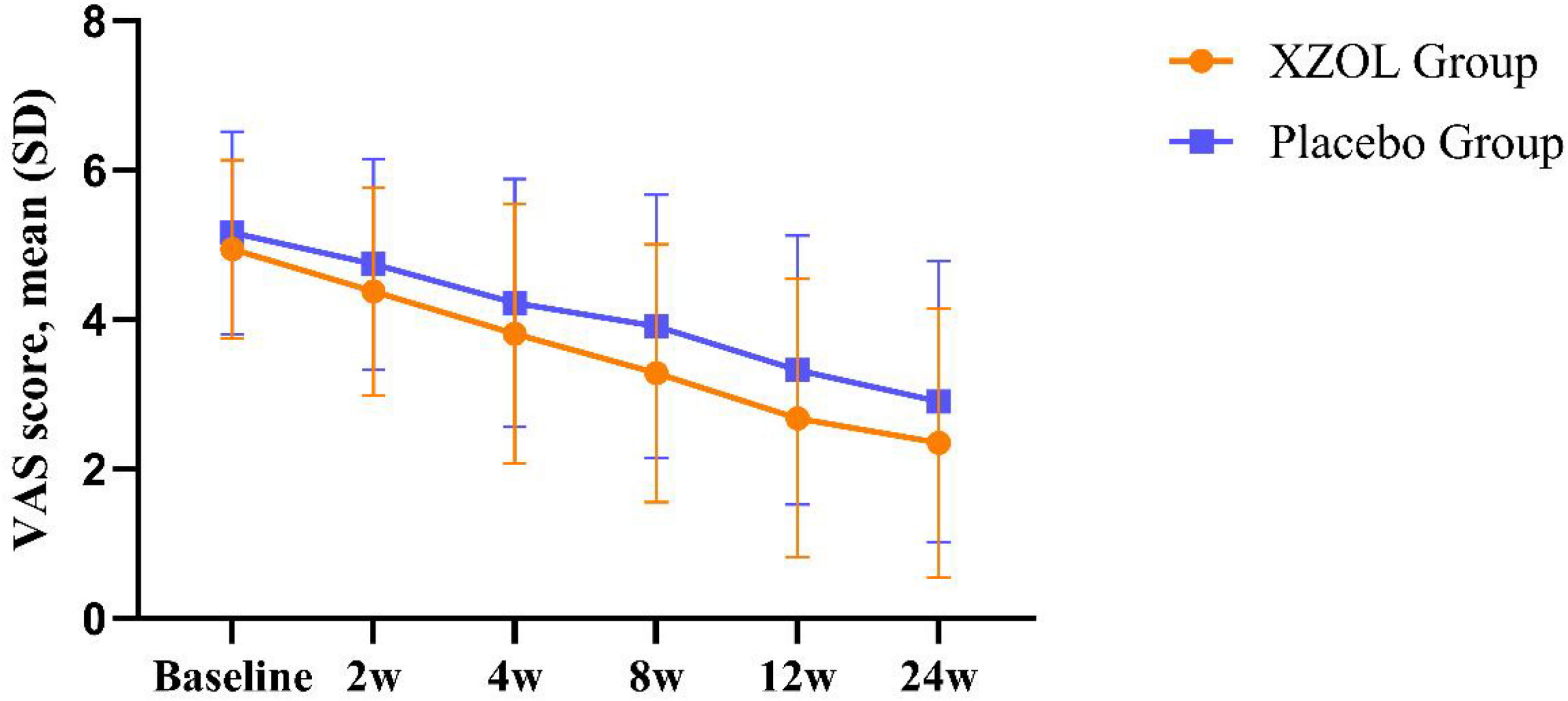
Difference in average angina pain intensity over time during the study by FAS. XZOL, Xuefu Zhuyu oral liquid; SD, standard deviation; FAS, full analysis set.

At week 12, patients in the XZOL group reported a greater reduction in angina pain intensity compared to the placebo group. The adjusted mean difference in VAS change from baseline to week 12 was –0.63 (95% CI: –1.12 to –0.14; *P* = 0.012) favoring XZOL (Table 2). The sensitivity analysis on the PPS yielded consistent and more pronounced results, with a mean difference of –1.11 (95% CI: –1.70 to –0.52; *P* < 0.001) between groups (Table 2). The center-by-treatment interaction was not statistically significant (Wald *x*^2^ = 2.90, df = 5, *P* = 0.716), indicating that the treatment effect of XZOL was consistent across study sites. Figure 2 illustrates the trends in mean VAS scores for both groups.

**TABLE 2:** Primary outcomes.

Primary outcome	FAS				PPS			
VAS score, mean (SD)	XZOL group (*n* = 74)	Placebo group (*n* = 74)	Adjusted difference (95% CI)	*P-*value^a^	XZOL group (*n* = 48)	Placebo group (*n* = 51)	Adjusted difference (95% CI)	*P-*value^a^
Baseline (t_0_)	4.95 (1.20)	5.16 (1.36)			4.97 (1.13)	5.20 (1.31)		
12 w (t_1_)	2.68 (1.86)	3.31 (1.83)			2.44 (1.80)	3.41 (1.89)		
t_1_–t_0_	–2.27 (1.71)	–1.85 (1.64)	–0.63 (–1.12 to –0.14)	0.012	–2.54 (1.83)	–1.79 (1.68)	–1.11 (–1.70 to –0.52)	< 0.001

CI, confidence interval; FAS, full analysis set; IQR, interquartile range; PPS, per-protocol analysis set; SD, standard deviation; VAS, visual analog scale; XZOL, Xuefu Zhuyu oral liquid.

^a^Statistical significance was calculated with a generalized linear model.

Subgroup analyses stratified by sex and age did not reveal significant differences. However, a more pronounced treatment effect was observed in the subgroup of patients with more severe baseline angina (CCS grade III). In this subgroup, patients receiving XZOL showed a significantly greater improvement in CCS grade at week 4 compared to placebo [mean scores: 5.20 (SD 1.47) vs. 6.68 (SD 0.83); mean difference –1.48 (95% CI: –3.31 to 0.35); *P* = 0.040] (Supplementary Tables 4–6 in Supplementary File 2).

### 3.2 Secondary outcomes

The beneficial effect of XZOL on angina pain was sustained through the 12-week follow-up. The adjusted mean difference in VAS change from baseline to week 24 was –0.47 (95% CI, –0.94 to –0.002; *P* = 0.049) in the FAS and –0.77 (95% CI, –1.33 to –0.20; *P* = 0.008) in the PPS, both favoring XZOL (Table 3). Although both groups experienced reductions in the frequency and duration of angina attacks, the differences between groups were not statistically significant. However, a key secondary finding was the significantly lower use of rescue nitroglycerin in the XZOL group. At week 12, 2.7% of patients in the XZOL group required rescue medication compared to 13.5% in the placebo group (*P* = 0.016). This difference persisted at week 24 (2.7% vs. 12.2%; *P* = 0.029). No significant between-group differences were observed for other secondary outcomes, including changes in CCS grade, QBS pattern, SAQ scores, EQ-5D-5L scores, or PSQI scores (Table 3).

**TABLE 3:** Secondary outcomes.

Secondary outcomes	FAS				PPS			
Outcomes	XZOL group (*n* = 74)	Placebo group (*n* = 74)	Mean difference (95% CI)	*P-*value^a^	XZOL group (*n* = 48)	Placebo group (*n* = 51)	Mean difference (95% CI)	*P-*value^a^
VAS at week 24, mean (SD)	2.35 (1.80)	2.88 (1.91)	NA	NA	2.15 (1.63)	3.06 (1.95)	NA	NA
Change at week 24, mean (SD)	–2.59 (1.61)	–2.28 (1.66)	–0.47 (–0.94 to –0.002)	0.049	–2.82 (1.64)	–2.14 (1.69)	–0.77 (–1.33 to –0.20)	0.008
Angina attack frequency, No. (%)								
Baseline								
≤ 1 Time/w	0	0	NA	0.447	0	0	NA	0.903
2–6 Times/w	62 (83.8)	65 (87.8)	NA		41 (85.4)	44 (86.3)	NA	
1–3 Times/d	10 (13.5)	9 (12.2)	NA		7 (14.6)	7 (13.7)	NA	
≥ 4 Times/d	2 (2.7)	0	NA		0	0	NA	
12 w								
≤ 1 Time/w	30 (40.5)	32 (43.2)	NA	0.761	26 (54.2)	25 (49.0)	NA	0.593
2–6 Times/w	40 (54.1)	38 (51.4)	NA		19 (39.6)	22 (43.1)	NA	
1–3 Times/d	4 (5.4)	4 (5.4)	NA		3 (6.3)	4 (7.8)	NA	
≥ 4 Times/d	0	0	NA		0	0	NA	
24w								
≤ 1 Time/w	44 (59.5)	48 (64.9)	NA	0.436	32 (66.7)	31 (60.8)	NA	0.617
2–6 Times/w	27 (36.9)	25 (33.8)	NA		14 (29.2)	19 (37.3)	NA	
1–3 Times/d	3 (4.1)	1 (1.4)	NA		2 (4.2)	1 (2.0)	NA	
≥ 4 Times/d	0	0	NA		0	0	NA	
Angina attack by duration, No. (%)								
Baseline								
No angina attack	0	0	NA	0.656	37 (77.1)	41 (80.4)	NA	0.742
≤ 5 min	55 (74.3)	58 (78.4)	NA		37 (77.1)	41 (80.4)	NA	
6–9 min	15 (20.3)	10 (13.5)	NA		8 (16.7)	6 (11.8)	NA	
≥ 10 min	4 (5.4)	6 (8.1)	NA		3 (6.3)	4 (7.8)	NA	
12w								
No angina attack	14 (18.9)	5 (6.8)	NA	0.345	12 (25.0)	5 (9.8)	NA	0.126
≤ 5 min	51 (68.9)	64 (86.5)	NA		32 (66.7)	42 (82.4)	NA	
6–9 min	8 (10.8)	3 (4.1)	NA		3 (6.4)	3 (5.9)	NA	
≥ 10 min	1 (1.4)	2 (2.7)	NA		1 (2.1)	1 (2.0)	NA	
24w								
No angina attack	20 (27.0)	13 (17.6)	NA	0.540	14 (29.2)	11 (21.6)	NA	0.704
≤ 5 min	46 (62.2)	57 (77.0)	NA		29 (60.4)	37 (72.5)	NA	
6–9 min	7 (9.5)	2 (2.7)	NA		4 (8.3)	2 (3.9)	NA	
≥ 10 min	1 (1.4)	2 (2.7)	NA		1 (2.1)	1 (2.0)	NA	
Use of rescue medicine, No. (%)								
Baseline	2 (2.7)	5 (6.8)	NA	0.247	0 (0)	3 (5.9)	NA	0.090
12 w	2 (2.7)	10 (13.5)	NA	0.016	1 (2.1)	7 (13.7)	NA	0.035
24 w	2 (2.7)	9 (12.2)	NA	0.029	1 (2.1)	8 (15.7)	NA	0.019
CCS grading for effort angina, no. (%)								
Baseline								
I	22 (29.7)	22 (29.7)	NA	0.906	14 (29.2)	14 (27.5)	NA	0.877
II	48 (64.9)	47 (63.5)	NA		32 (66.7)	35 (68.6)	NA	
III	4 (5.4)	5 (6.8)	NA		2 (4.2)	2 (3.9)	NA	
12 w								
I	42 (56.8)	43 (58.1)	NA	0.927	25 (52.1)	27 (52.9)	NA	0.930
II	31 (41.9)	29 (39.2)	NA		22 (45.8)	23 (45.1)	NA	
III	1 (1.4)	2 (2.7)	NA		1 (2.1)	1 (2.0)	NA	
24 w								
I	47 (63.5)	48 (64.9)	NA	0.916	28 (58.3)	31 (60.8)	NA	0.806
II	26 (35.1)	24 (32.4)	NA		19 (39.6)	19 (37.3)	NA	
III	1 (1.4)	2 (2.7)	NA		1 (2.1)	1 (2.0)	NA	
QBS pattern changes								
Baseline	74 (100)	74 (100)	NA	1.000	48 (100)	51 (100)	NA	1.000
12 w	71 (95.9)	74 (100)	NA	0.245	46 (95.8)	51 (100)	NA	0.233
24 w	69 (93.2)	72 (97.3)	NA	0.442	45 (93.8)	49 (96.1)	NA	0.672
SAQ score in PL, mean (SD)								
Baseline	59.30 (13.11)	59.18 (13.51)	0.12 (–4.15 to 4.38)	0.957	59.44 (11.39)	58.69 (13.51)	0.75 (–4.25 to 5.75)	0.766
12 w	63.19 (11.74)	63.71 (11.94)	–0.52 (–4.32 to 3.27)	0.785	64.37 (10.50)	64.39 (11.65)	–0.02 (–4.46 to 4.41)	0.991
24 w	65.14 (11.40)	65.54 (11.73)	–0.40 (–4.11 to 3.31)	0.831	65.42 (10.28)	66.36 (11.28)	–0.94 (–5.26 to 3.37)	0.665
SAQ score in AS, mean (SD)								
Baseline	52.30 (17.41)	51.97 (14.58)	0.33 (–4.82 to 5.48)	0.900	51.56 (15.82)	50.49 (13.68)	1.07 (–4.82 to 6.96)	0.719
12 w	76.12 (19.18)	74.03 (20.47)	2.09 (–4.27 to 8.44)	0.518	76.19 (21.08)	71.25 (21.50)	4.94 (–3.56 to 13.44)	0.252
24 w	67.15 (20.78)	72.20 (20.72)	–5.05 (–11.70 to 1.61)	0.136	66.15 (22.77)	72.55 (21.36)	–6.40 (–15.21 to 2.40)	0.152
SAQ score in AF, mean (SD)								
Baseline	70.13 (11.37)	69.47 (12.95)	0.66 (–3.25 to 4.56)	0.740	71.04 (11.53)	69.80 (12.73)	1.24 (–3.62 to 6.09)	0.614
12 w	83.96 (11.58)	82.16 (11.98)	1.81 (–1.97 to 5.58)	0.346	85.53 (10.58)	83.38 (11.81)	2.14 (–2.34 to 6.63)	0.345
24 w	85.98 (11.42)	84.57 (11.83)	1.41 (–2.32 to 5.13)	0.457	86.25 (11.42)	84.71 (10.46)	1.54 (–2.82 to 5.91)	0.484
SAQ score in TS, mean (SD)								
Baseline	65.17 (18.12)	69.04 (16.73)	–3.87 (–9.46 to 1.72)	0.173	62.75 (17.30)	68.40 (18.03)	–5.65 (–12.71 to 1.41)	0.115
12 w	73.20 (13.28)	74.57 (13.47)	–1.37 (–5.66 to 2.92)	0.528	72.29 (13.14)	75.60 (13.79)	–3.30 (-8.68 to 2.08)	0.226
24 w	75.88 (13.77)	75.49 (14.66)	0.39 (–4.17 to 4.95)	0.867	75.74 (14.34)	76.12 (15.31)	–0.39 (–6.32 to 5.54)	0.897
Baseline	57.02 (18.91)	61.29 (16.48)	–4.28 (–9.96 to 1.41)	0.139	57.47 (18.53)	61.44 (16.66)	–3.97 (–11.00 to 3.05)	0.264
12 w	68.43 (14.93)	70.14 (14.61)	–1.71 (–6.45 to 3.02)	0.476	68.56 (14.33)	70.47 (14.19)	–1.91 (–7.60 to 3.78)	0.508
24 w	70.16 (14.88)	71.65 (15.65)	–1.49 (–6.38 to 3.41)	0.550	70.83 (14.89)	73.20 (16.27)	–2.37 (–8.60 to 3.86)	0.452
EQ-5D-5L score, mean (SD)								
Baseline	0.87 (0.14)	0.89 (0.12)	–0.01 (–0.05 to 0.03)	0.580	0.89 (0.08)	0.90 (0.12)	0.00 (–0.04 to 0.04)	0.929
12 w	0.93 (0.06)	0.93 (0.08)	0.00 (–0.02 to 0.03)	0.759	0.94 (0.05)	0.94 (0.08)	0.01 (–0.02 to 0.04)	0.547
24 w	0.93 (0.06)	0.93 (0.08)	–0.001 (–0.02 to 0.02)	0.910	0.94 (0.05)	0.94 (0.09)	0.00 (–0.02 to 0.03)	0.738
EQ-5D-5L VAS score, mean (SD)								
Baseline	72.64 (11.50)	71.89 (13.23)	0.74 (–3.29 to 4.77)	0.716	71.96 (12.12)	72.49 (13.56)	–0.53 (–5.66 to 4.59)	0.838
12 w	81.62 (8.96)	79.81 (10.64)	1.81 (–1.38 to 5.01)	0.264	81.94 (8.55)	79.99 (10.86)	1.95 (–1.94 to 5.83)	0.326
24 w	83.26 (9.18)	80.69 (10.42)	2.56 (–0.63 to 5.76)	0.114	83.21 (9.32)	80.73 (11.14)	2.48 (–1.61 to 6.57)	0.234
PSQI score, mean (SD)								
Baseline	11.82 (2.89)	11.97 (2.78)	–0.15 (–1.07 to 0.78)	0.755	11.82 (2.92)	11.72 (2.64)	0.11 (–1.00 to 1.22)	0.848
12 w	4.18 (2.88)	4.94 (3.31)	–0.75 (–1.76 to 0.25)	0.141	4.05 (2.73)	5.00 (3.51)	–0.95 (–2.21 to 0.31)	0.139
24 w	3.74 (2.75)	4.26 (3.22)	–0.52 (–1.50 to 0.45)	0.289	3.79 (2.66)	4.29 (3.43)	–0.50 (–1.73 to 0.73)	0.419

AF, angina frequency; AS, angina stability; CCS, Canadian Cardiovascular Society; CI, confidence interval; DP, disease perception; EQ-5D-5L, EuroQol-5-Dimensions-5-Level; FAS, full analysis set; NA, not applicable; PL, physical limitation; PPS, per-protocol analysis set; PSQI, Pittsburgh sleep quality index; QBS, Qi stagnation and Blood stasis; SAQ, Seattle Angina Questionnaire; TS, treatment satisfaction; VAS, visual analog scale; XZOL, Xuefu Zhuyu oral liquid; Zung-SAS, Zung Self-rating Anxiety Scale; Zung-SDS, Zung Self-rating Depression Scale.

^a^Statistical significance was calculated based on data distribution. *P-*values were calculated with a Mann-Whitney *U*-test between the 2 groups for angina attack frequency; angina attack duration; use of rescue medicine and CCS grading for effort angina. *P-*values for SAQ; EQ-5D-5L index; EQ-5D-5L VAS and PSQI were calculated with an independent-samples *t*-test between the 2 groups. *P-*values for *Qi* stagnation and *Blood* stasis pattern changes were calculated with a chi-square test.

### 3.3 Adverse events

Safety was assessed in all 152 randomized participants. A total of 16 AEs were reported, with an identical incidence in both groups: 8 events (10.5%) in the XZOL group and 8 events (10.5%) in the placebo group (*P* = 1.000) (Table 4). Most AEs were mild and self-limiting.

**TABLE 4:** Adverse events for both groups.

AEs	Participants; no. (%) (*N* = 148)		*P-*value^a^
	XZOL group (*n* = 76)	Placebo group (*n* = 76)	
Overall	8 (10.5)	8 (10.5)	1.000
Severe AEs			
Unstable angina pectoris	1 (1.3)	0 (0)	
Other AEs			
Abnormal urine routine test	6 (7.9)	7 (9.2)	
Abnormal liver function	1 (1.3)	0 (0)	
Edema	1 (1.3)	0 (0)	
Stomachache	0 (0)	1 (1.3)	
Discontinuation due to AEs			
Yes	0 (0)	1 (1.3)	

AEs, adverse events; XZOL, Xuefu Zhuyu oral liquid.

^a^Statistical significance was calculated with a Fisher’s exact test.

One SAE, an episode of unstable angina, occurred in a participant in the XZOL group. The event was adjudicated by the DSMC as unrelated to the study drug, and the patient made a full recovery. One participant in the placebo group discontinued the trial due to stomachache, which was considered possibly related to the intervention. One case of abnormal liver function in the XZOL group was deemed possibly related to the study drug; the patient recovered fully after appropriate management and completed the trial.

## 4 Discussion

This multicenter, double-blind, placebo-controlled trial found that a 12-week course of XZOL, when added to standard antianginal therapy, resulted in a statistically significant reduction in angina pain intensity in patients with SA. This benefit was sustained for 12 weeks after treatment cessation. Although statistically robust, the observed effect size for the primary outcome did not meet our prespecified superiority margin of 1.7. This suggests that the average therapeutic benefit of XZOL on pain intensity, while measurable, may be modest in the overall study population. Importantly, the safety profile of XZOL was favorable, with an AE incidence comparable to that of placebo. To our knowledge, this is the largest and most rigorous trial to date evaluating the efficacy and safety of XZOL as an adjunctive therapy for SA over a 24-week period.

Beyond the primary outcome, several other findings support the clinical value of XZOL. First, the subgroup analysis suggested a more pronounced effect in patients with more severe baseline symptoms (CCS grade III), indicating that this population may derive greater benefit from XZOL. Second, the significantly lower use of rescue nitroglycerin in the XZOL group provides objective evidence of improved symptom control. Our trial also demonstrated a favorable safety profile for XZOL, with identical overall AE incidence in the XZOL and placebo groups (10.5%). Most AEs were mild and self-limiting. These findings suggest XZOL is well tolerated as an adjunct to standard therapy. Although all participants continued their baseline cardiovascular medications, no clinically significant interactions or safety signals were observed during the trial. Nonetheless, clinical vigilance is warranted, and dedicated pharmacokinetic studies are needed to formally assess these potential risks.

We selected the VAS score as the primary outcome because it directly measures the patient’s symptomatic experience and effectively assesses the pain in angina pectoris (27–31). As a validated and widely used patient-reported outcome (PRO) in pain research, the VAS provides a clinically meaningful assessment of treatment benefit (32, 33), consistent with recommendations from initiatives like IMMPACT (34). Therefore, despite the superiority margin not being met, the statistically significant reduction in VAS scores in the XZOL group compared to placebo indicates a genuine, albeit modest, improvement in patients’ symptomatic burden.

Our findings build on and strengthen the existing evidence for Xuefu Zhuyu decoction in the management of SA. While a previous systematic review of 14 RCTs reported that adjunctive Xuefu Zhuyu decoction was effective for relieving symptoms and improving objective markers such as electrocardiogram (ECG) and high-density lipoprotein cholesterol (HDL-C) levels, its conclusions were undermined by significant methodological limitations (13). The included trials were predominantly short-term (4–8 weeks) and of low methodological quality, which compromises the reliability of their findings and underscores a critical need for more robust, long-term evidence—a gap our current study aims to address. Preclinical evidence suggests that Xuefu Zhuyu decoction exerts its therapeutic effects through multiple biological pathways. Animal studies have demonstrated that it increases serum levels of superoxide dismutase and interleukin-10, while reducing levels of inflammatory mediators such as soluble intercellular adhesion molecule-1, endothelin-1, and interleukin-6 (35). Another proposed mechanism involves modulation of platelet-activating factor, which helps mitigate the development of coronary atherosclerosis (36). Research has also shown that it improves hemorheological parameters, restores microcirculatory flow in blood stasis models, and attenuates myocardial fibrosis (37) and the oxidative damage to myocardial cells (38). Taken together, these preclinical findings offer a mechanistic basis for the therapeutic effects of Xuefu Zhuyu preparation in ameliorating multiple symptoms in patients with SA, although the precise molecular mechanisms warrant further investigation.

This study possesses several notable strengths. Primarily, it was a well-designed, multicenter, large-scale clinical trial that offers robust evidence supporting the use of XZOL alongside standard antianginal therapies for SA. Additionally, the extended duration of the study, including a 12-week follow-up period, allowed for the evaluation of the sustained effects of XZOL. Despite the unprecedented challenges posed by the COVID-19 pandemic, our implementation of continuous monitoring helped mitigate its impact on patient adherence, which remained relatively high among participants who continued the study.

Despite its strengths, this study has several important limitations. First, the reliance on a subjective primary outcome (VAS for pain) makes the results susceptible to a significant placebo effect. The substantial improvement observed in the placebo group likely contributed to the treatment effect failing to meeting the prespecified superiority margin. Future trials should incorporate objective functional measures, such as exercise tolerance testing or myocardial perfusion imaging, to corroborate these symptomatic findings. Second, maintaining effective blinding was a challenge. The distinctive organoleptic properties (taste and smell) of XZOL, a common issue in herbal medicine trials, may have allowed some participants to guess their treatment assignment, potentially introducing reporting bias. Third, the generalizability of our findings is limited. The trial was conducted exclusively in a Chinese population, and all participants were required to have a specific CM pattern QBS). Consequently, these results may not be directly applicable to other ethnic groups or to the broader population of patients with SA who do not fit this diagnostic pattern.

## 5 Conclusion

In this randomized controlled trial, a 12-week course of XZOL as an adjunctive therapy significantly reduced angina pain intensity compared to placebo in patients with SA. These results suggest that XZOL may be a valuable complementary treatment option for managing symptoms in this patient population.

## Data Availability

The raw data supporting the conclusions of this article will be made available by the authors, without undue reservation.
