# Supplementary material for: Effects of Xuefu Zhuyu oral liquid as adjunctive treatment for stable angina: a randomized controlled trial
Source: Front Med (Lausanne). 2026 Apr 29;13:1787481. doi: 10.3389/fmed.2026.1787481 (PMC13167926; doi:10.3389/fmed.2026.1787481)
Supplement: Supplementary file 1 [file Supplementary_file_1.docx]

**Detailed information on two interventions**

1. The dose and scientific names of all ingredients in Xuefu zhuyu oral liquid (XZOY)

Online Table 1. The dose and scientific names of all ingredients in XZOY

| Chinese name | English name | Latin name | Dose* |
| --- | --- | --- | --- |
| Chaihu | Bupleuri Radix | *Bupleurum chinense DC.* | 17g |
| Danggui | Angelicae Sinensis Radix | *Angelica sinensis (Oliv.) Diels* | 50g |
| Shengdihuang | Rehmanniae Radix | *Rehmannia glutinosa Libosch.* | 50g |
| Chishao | Paeoniae Radix Rubra | *Paeonia lactiflora Pall* | 33g |
| Honghua | Carthami Flos | *Carthamus tinctorius L.* | 50g |
| Taoren | Persicae Semen | *Prunus persica (L.)Batsch* | 67g |
| Zhiqiao | Aurantii Fruxtus | *Citrus aurantium L.* | 33g |
| Gancao | Glycyrrhizae Radix et Rhizoma | *Glycyrrhiza uralensis Fisch.* | 17g |
| Chuanxiong | Chuangxiong Rhizoma | *Ligusticum chuanxiong Hort.* | 25g |
| Niuxi | Achyranthis Bidentatae Radix | *Achyranthes bidentata Bl.* | 50g |
| Jiegeng | Platycodonis Radix | *Platycodon grandiflorum (Jacq.) A.DC.* | 25g |

* The dose of each drug in 1000ml liquid.

2. Pharmaceutical production processes of XZOY

XZOY was manufactured in strict accordance with the standards of the Chinese Pharmacopoeia (2020), and the process strictly abides by the good manufacturing practices (GMP). The outline is as following:

There are 11 herbal medicines in the liquid, Bupleuri Radix, Angelicae Sinensis Radix, Aurantii Fruxtus, and Chuangxiong Rhizoma distilled to extract aromatic water, set aside; medicine residue and other herbal medicines were decocted 3 times, 2 hours each time, combine decoction, filter, and filtrate concentrated to a relative density of about 1.10 (60°C), add ethanol to make the alcohol content up to 60%, refrigerate for 24 hours, filter, and recycle the filtrate to the ethanol until no alcohol taste, add 100 g sucrose, 200 g honey, 0.5 g potassium sorbate and the above aroma water, stir, add water to 1000 ml and mix well, adjust the pH to 5.0, refrigerate, filter, fill, sterilize, and get ready.

Online Table 2. The dose and scientific names of all ingredients in placebo

| English name | Dose^#^ |
| --- | --- |
| Honey | 100g |
| White granulated sugar | 200g |
| Fried white sugar | 19g |
| Fried brown sugar | 7g |
| Bitterant | 0.05g |
| Potassium sorbate | 1g |
| Sodium benzoate | 1g |
| Ethanol (95%) | 50ml |
| Ginseng essence | 0.1g |

^#^The above drugs are made into 1000g finished products.


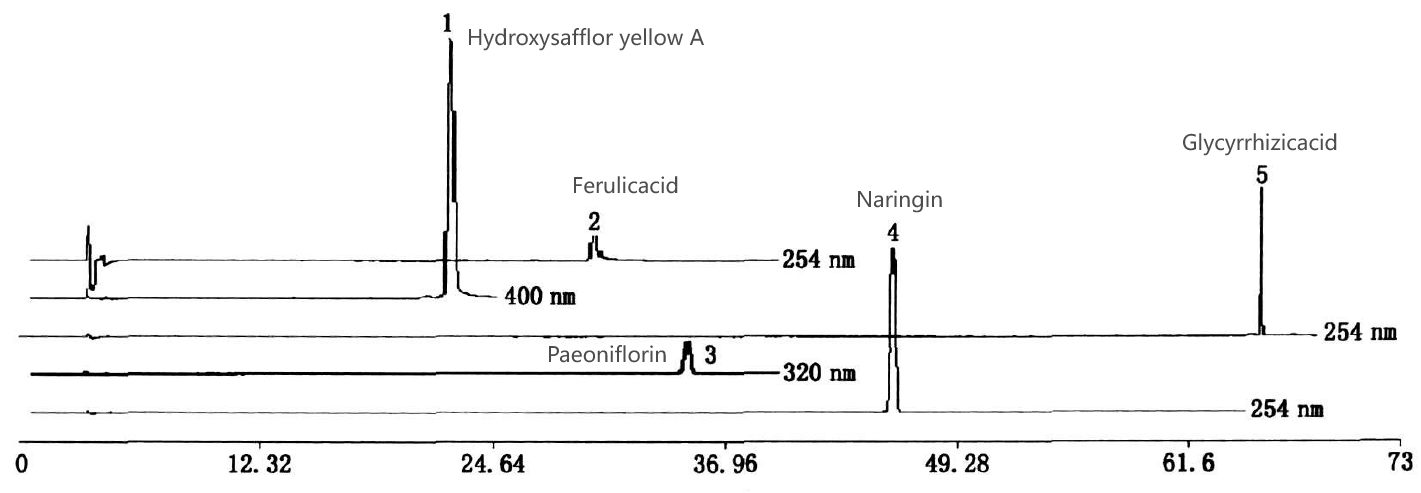


Online Figure 1. Fingerprint information of XZOY: the obtained finger pattern includes two characteristic peaks.
