# Supplementary material for: Effects of Xuefu Zhuyu oral liquid as adjunctive treatment for stable angina: a randomized controlled trial
Source: Front Med (Lausanne). 2026 Apr 29;13:1787481. doi: 10.3389/fmed.2026.1787481 (PMC13167926; doi:10.3389/fmed.2026.1787481)
Supplement: Supplementary file 2 [file Supplementary_file_2.docx]

**1. Study Inclusion and Exclusion Criteria (Online Table 1, 2, and 3)**

1.1. Diagnostic criteria for stable angina (SA) due to CHD.

According to the Guideline for the Diagnosis and Treatment of Patients with Stable Ischemic Heart Disease issued by the Chinese Society of Cardiology in 2018 and the Guideline Update for the Management of Patient with Chronic Stable Angina Pectoris released by the American Heart Association/ American College of Cardiology (ACC/AHA) in 2002, the diagnostic criteria for SA due to CHD are as follows:

(1) Anginal pain with the following four characteristics: (a) Location: the anginal pain usually occurs in the substernal chest; atypical locations include the epigastrium, the lower jaw or teeth, shoulder blades, arms, wrists, or fingers. (b) Property: the anginal pain generally presents with pressure, tightness or heaviness, sometimes strangling, constricting, or burning. It is occasionally accompanied by shortness of breath or other nonspecific symptoms such as fatigue or faintness, nausea, burning, restlessness, or a sense of impending doom. (c) Duration: the anginal pain usually lasts less than 10 minutes. (d) Predisposing and relieving factors: the anginal pain can be triggered by physical exertion such as walking up an incline, walking against a breeze, ingesting a heavy meal, or cold weather. It can also be triggered by emotional stress. It usually resolves within few minutes by rest or with sublingual nitrates.

(2) The anginal pain is present for at least 2 months without changes in frequency, characteristics, or triggering factors

1.2 Grading of Angina Pectoris.

The Grading of Angina of Effort by the Canadian Cardiovascular Society (CCS) will be used as a grading system to quantify SA severity (OnlineTable 2).

1.3 Qi-Stagnation and Blood-Stasis Pattern (QBP) Diagnosis. QBP in CM will be diagnosed by a qualified CM physician according to the diagnosis scale for Qi-stagnation and blood-stasis syndrome (DSQBS). The cutoff point for the DSQBS score is 20 points for QBP diagnosis (eTable 3).

Online Table 1 Study inclusion and exclusion criteria

| Criteria | Details |
| --- | --- |
| Eligibility | The study’s eligibility was assessed in a prescreening, ensuring participant integrity, confidentiality, and anonymity. The following inclusion and exclusion criteria were employed for the study. |
| Inclusion Criteria | (1) Meeting the diagnostic criteria of SA and QBP in CM  (2) Age between 30 and 75 years  (3) Males with any of the following conditions and females with b, c, d, or e: (a) Positive second quantity exercise treadmill testing (b) Positive radionuclide exercise test (c) Suffered from myocardial infarction for more than 3 months (d) Undergone percutaneous coronary intervention (PCI) for more than 12 months (e) Presentation of a stenosis ≥50% in at least one main coronary artery or its larger branches in either coronary angiography or coronary computer tomographic angiography  (4) The CCS grading of effort angina is either I, II, or III  (5) The frequency of angina attack ≥ 2 weekly and ≤6 daily  (6) Visual analogue scale (VAS) used to measure average intensity pain of angina ≥ 3 cm over the past 2 weeks  (7) Signed informed consent |
| Exclusion Criteria | (1) Suffering from severe heart disease including unstable angina pectoris, severe arrhythmias (e.g., rapid atrial fibrillation, atrial flutter, paroxysmal ventricular tachycardia, Mobitz II second degree atrioventricular block, and third-degree atrioventricular block), and severe cardiopulmonary insufficiency (e.g., cardiac function grade IV and severely abnormal pulmonary function)  (2) Poorly controlled hypertension (systolic blood pressure ≥ 160 mmHg or diastolic blood pressure ≥ 100 mmHg after treatment)  (3) Planning to undergo coronary artery bypass grafting (CABG) or PCI during the trial  (4) Suffering from any disease that triggers chest pain, such as other heart diseases, neurosis, menopausal syndrome, spondylosis disease, or gastroesophageal reflux disease (GERD)  (5) Suffering from severe liver or renal dysfunction (creatinine value over the upper limit for normal reference, alanine aminotransferase, aspartate aminotransferase, or total bilirubin value over 1.5 times the upper limit for normal reference)  (6) Suffering from severe primary diseases, such as diseases of the respiratory, digestive, urinary, or hemopoietic system, or tumors  (7) Zung Self-rating Anxiety Scale (Zung-SAS) > 59 or Zung Self-rating Depression Scale (Zung-SDS) > 62  (8) Gravida, lactating women, or individuals planning to conceive  (9) Failing to cooperate with the investigator to participate in the trial (e.g., blindness, deafness, dumbness, intellectual disability, and mental disability)  (10) Participation in other clinical trials within the last 3 months  (11) Having allergic constitution or being allergic to the known component of the study drug  (12) Not being suitable for the trial, as judged by an investigator |

Online Table 2 Grading of angina of effort by the CCS

| Grading | Details |
| --- | --- |
| Ⅰ | Ordinary activity, such as walking and ascending stairs, does not cause angina; angina with strenuous or rapid or prolonged exertion at work or recreation |
| Ⅱ | Slight limitation of ordinary activity; angina walking or ascending stairs rapidly, walking or ascending stairs after meals, or in the cold, wind or under emotional stress, or only during the first few hours awake, walking more than 200 m on level ground or ascending more than one flight of ordinary stairs at a normal pace under normal conditions |
| Ⅲ | Marked limitations in ordinary physical activity; angina when walking 100–200 m on level ground or one flight of stairs under normal conditions and at a normal pace |
| Ⅳ | Inability to partake in any physical activity without discomfort—angina syndrome may be present at rest |

Online Table 3 Diagnosis scale for QBP

| Symptoms and signs | Yes | No | Score |
| --- | --- | --- | --- |
| Pain^⁎^ | 9 | 0 | —— |
| Irritability/ depression | 16 | 0 | —— |
| Distending pain | 2 | 0 | —— |
| Scurry pain | 6 | 0 | —— |
| Chest distress | 0.5 | 0 | —— |
| Lumps in body | 7 | 0 | —— |
| Petechia in the tongue | 4 | 0 | —— |
| Purplish tongue | 1 | 0 | —— |
| Uneven pulse | 4 | 0 | —— |
| Deep pulse | 2 | 0 | —— |
| If the sum score ≥20, QBP can be diagnosed. |  | Score: | —— |

Notes: ^⁎^Pain including stomachache, abdominal pain, low back pain, dysmenorrhea, breast pain, and limb pain, etc. QBP: qi-stagnation and blood-stasis pattern.

**2.** Online Table 4 VAS score subgroup analyses by Gender

|  | FAS | | | |
| --- | --- | --- | --- | --- |
| Gender | XFZY group | Placebo group | Mean difference (95% CI) | *P* Value |
| Female | n = 21 | n = 26 |  |  |
| Baseline | 5.06 (1.37) | 5.52 (1.42) | -0.46 (-1.29 to 0.37) | 0.267 |
| 2 w | 4.56 (1.38) | 4.99 (1.34) | -0.43 (-1.24 to 0.37) | 0.285 |
| 4 w | 4.25 (1.61) | 4.24 (1.89) | 0.01 (-1.04 to 1.06) | 0.986 |
| 8 w | 3.55 (1.74) | 4.20 (1.68) | -0.66 (-1.66 to 0.35) | 0.196 |
| 12 w | 2.95 (1.85) | 3.30 (2.01) | -0.35 (-1.49 to 0.80) | 0.547 |
| 24 w | 2.55 (1.92) | 2.97 (1.95) | -0.42 (-1.57 to 0.72) | 0.458 |
| Male | n = 53 | n = 48 |  |  |
| Baseline | 4.90 (1.13) | 4.96 (1.30) | -0.07 (-0.55 to 0.41) | 0.784 |
| 2 w | 4.30 (1.40) | 4.60 (1.44) | -0.30 (-0.86 to 0.26) | 0.295 |
| 4 w | 3.64 (1.77) | 4.21 (1.54) | -0.58 (-1.23 to 0.08) | 0.085 |
| 8 w | 3.18 (1.73) | 3.75 (1.80) | -0.58 (-1.27 to 0.12) | 0.105 |
| 12 w | 2.57 (1.88) | 3.31 (1.74) | -0.74 (-1.46 to -0.02) | 0.043 |
| 24 w | 2.28 (1.77) | 2.84 (1.91) | -0.56 (-1.29 to 0.16) | 0.130 |

**3.** Online Table 5 VAS score subgroup analyses by Age

|  | FAS | | | |
| --- | --- | --- | --- | --- |
| Age | XFZY group | Placebo group | Mean difference (95% CI) | *P* Value |
| Age≤65 | n = 44 | n = 39 |  |  |
| Baseline | 4.88 (1.25) | 5.03 (1.31) | -0.28 (-0.92 to 0.37) | 0.454 |
| 2 w | 4.31 (1.47) | 4.67 (1.27) | -0.38 (-1.10 to 0.34) | 0.707 |
| 4 w | 3.91 (1.62) | 4.04 (1.53) | -0.81 (-1.73 to 0.11) | 0.773 |
| 8 w | 3.14 (1.82) | 3.64 (1.72) | -0.70 (-1.55 to 0.15) | 0.253 |
| 12 w | 2.63 (1.93) | 3.07 (1.68) | -0.75 (-1.70 to 0.21) | 0.464 |
| 24 w | 2.30 (1.82) | 2.76 (1.76) | -0.52 (-1.51 to 0.48) | 0.342 |
| Age＞65 | n = 30 | n = 35 |  |  |
| Baseline | 5.03 (1.13) | 5.31 (1.42) | -0.28 (-0.92 to 0.37) | 0.395 |
| 2 w | 4.48 (1.29) | 4.82 (1.57) | -0.34 (-1.06 to 0.38) | 0.350 |
| 4 w | 3.66 (1.92) | 4.43 (1.79) | -0.77 (-1.69 to 0.15) | 0.101 |
| 8 w | 3.49 (1.59) | 4.21 (1.78) | -0.72 (-1.56 to 0.11) | 0.092 |
| 12 w | 2.75 (1.79) | 3.56 (1.97) | -0.81 (-1.75 to 0.12) | 0.089 |
| 24 w | 2.43 (1.80) | 3.02 (2.09) | -0.59 (-1.57 to 0.39) | 0.231 |

**4.** Online Table 6 VAS score subgroup analyses by CCS

|  | FAS | | | |
| --- | --- | --- | --- | --- |
| CCS | XFZY group | Placebo group | Mean difference (95% CI) | *P* Value |
| Ⅰ & Ⅱ | n = 70 | n = 69 |  |  |
| Baseline | 4.93 (1.17) | 5.02 (1.26) | -0.09 (-0.50 to 0.32) | 0.662 |
| 2 w | 4.35 (1.38) | 4.58 (1.30) | -0.23 (-0.69 to 0.22) | 0.305 |
| 4 w | 3.73 (1.73) | 4.05 (1.56) | -0.31 (-0.87 to 0.24) | 0.263 |
| 8 w | 3.27 (1.71) | 3.72 (1.66) | -0.46 (-1.02 to 0.11) | 0.112 |
| 12 w | 2.66 (1.81) | 3.13 (1.72) | -0.46 (-1.05 to 0.13) | 0.124 |
| 24 w | 2.31 (1.76) | 2.70 (1.80) | -0.40 (-0.99 to 0.20) | 0.190 |
| Ⅲ | n = 4 | n = 5 |  |  |
| Baseline | 5.20 (1.80) | 7.10 (1.26) | -1.90 (-4.31 to 0.51) | 0.099 |
| 2 w | 4.93 (1.66) | 6.94 (0.99) | -2.02 (-4.11 to 0.08) | 0.098 |
| 4 w | 5.20 (1.47) | 6.68 (0.83) | -1.48 (-3.31 to 0.35) | 0.040 |
| 8 w | 3.60 (2.30) | 6.52 (0.65) | -2.92 (-5.43 to -0.41) | 0.121 |
| 12 w | 2.93 (3.07) | 5.78 (1.65) | -2.86 (-6.60 to 0.89) | 0.134 |
| 24 w | 3.18 (2.62) | 5.38 (1.85) | -2.21 (-5.72 to 1.31) | 0.502 |
