# Supplementary material for: Effects of Xuefu Zhuyu oral liquid as adjunctive treatment for stable angina: a randomized controlled trial
Source: Front Med (Lausanne). 2026 Apr 29;13:1787481. doi: 10.3389/fmed.2026.1787481 (PMC13167926; doi:10.3389/fmed.2026.1787481)
Supplement: Supplementary file 3 [file Supplementary_file_3.docx]

**Multiple Imputation Procedure**

Missing data for post-baseline endpoints in the modified Intention-to-Treat (mITT) and Per-Protocol Set (PPS) analyses were addressed using multiple imputation (MI). This procedure was conducted under the guiding assumption that the data were Missing At Random (MAR), meaning that the probability of a value being missing depends on observed data but not on the unobserved missing value itself. A multivariate imputation model was constructed using automatic model selection. The model included the treatment group, baseline value of the endpoint, and all available post-baseline measurements. A total of five imputed datasets were generated. Following imputation, the primary analysis was performed independently on each of the five complete datasets. Finally, these five sets of results were combined into a single, valid statistical inference.
